# Long-term survival from gastrocolic fistula secondary to adenocarcinoma of the transverse colon

**DOI:** 10.1186/1477-7819-3-9

**Published:** 2005-02-10

**Authors:** Matthew J Forshaw, Jamasp K Dastur, Kothandaraman Murali, Michael C Parker

**Affiliations:** 1 Department of Surgery, Darent Valley Hospital, Dartford, Kent, DA2 8DA, UK

## Abstract

**Background:**

Gastrocolic fistula is a rare presentation of both benign and malignant diseases of the gastrointestinal tract. Malignant gastrocolic fistula is most commonly associated with adenocarcinoma of the transverse colon in the Western World. Despite radical approaches to treatment, long-term survival is rarely documented.

**Case presentation:**

We report a case of a 24-year-old woman who presented with the classic triad of symptoms associated with gastrocolic fistula. Radical *en-bloc *surgery and adjuvant chemotherapy were performed. She is still alive ten years after treatment.

**Conclusions:**

Gastrocolic fistula is an uncommon presentation of adenocarcinoma of the transverse colon. Radical *en-bloc *surgery with adjuvant chemotherapy may occasionally produce long-term survival.

## Background

Gastrocolic fistula is a rare complication of both benign and malignant diseases of the gastrointestinal tract [1-6]. In the Western World, adenocarcinoma of the transverse colon is the commonest cause of a fistulous connection between the stomach and the colon with a reported incidence of 0.3–0.4% in operated cases [3,4]. Despite radical en-bloc surgery, these patients usually have a poor prognosis [5,6]. Long-term survival for these patients is rarely reported [5].

The authors report a 24-year-old woman who presented with a gastrocolic fistula secondary to an adenocarcinoma of the transverse colon and describe her treatment and long-term follow up.

## Case presentation

A 24-year-old woman presented to the surgical clinic with epigastric pain, feculent vomiting and post-prandial diarrhoea of three months duration; she had lost over one stone in weight. She was previously healthy and was not taking any regular medications. There was no history of peptic ulcer disease, inflammatory bowel disease, trauma or previous abdominal surgery. She had been investigated two years previously by a gastroenterologist for intermittent left-sided abdominal pain at which time the clinical examination and blood tests were normal. Irritable bowel syndrome had been diagnosed, although no colonic imaging was performed. Both her maternal grandfather and great-grandfather had suffered from colonic cancer.

**Figure 1:**
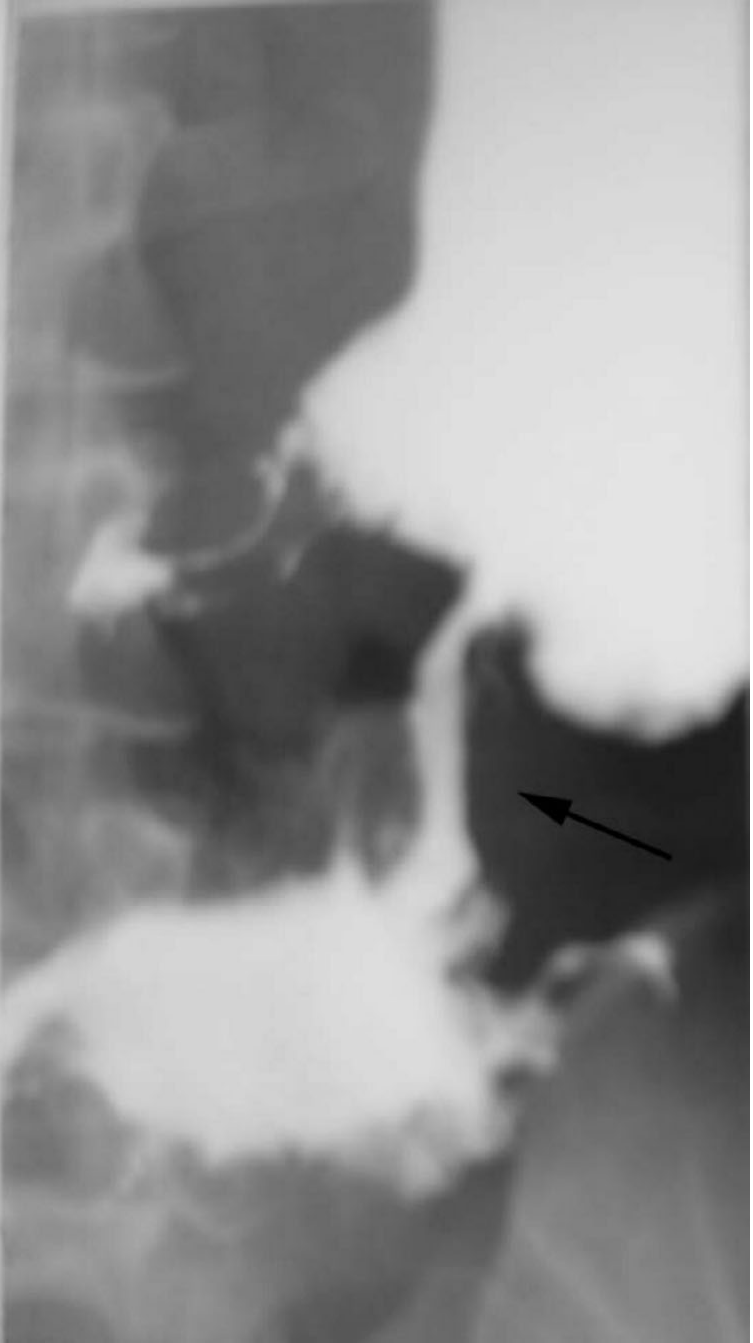
Barium meal demonstrating fistulous connection between greater curvature of the stomach and the distal half of the transverse colon (arrowed).

An initial ultrasound scan of the abdomen revealed thickened bowel in the right upper quadrant with a dilated duodenum. A barium meal and follow through was then performed: this demonstrated a mucosal abnormality on the greater curvature of the stomach with a fistulous tract into the transverse colon (Figure 1). Barium enema and colonoscopy were not performed. The presence of a mucosal abnormality on the greater curvature of the stomach was confirmed on upper gastrointestinal endoscopy although initial biopsies revealed no evidence of a malignant neoplasm. Her blood tests revealed: haemoglobin 9.5 g/dl, mean cell volume 71.6 fl and a white cell count 20.2 × 10^9^/l; urea, electrolytes and liver function tests were all normal.

**Figure 2:**
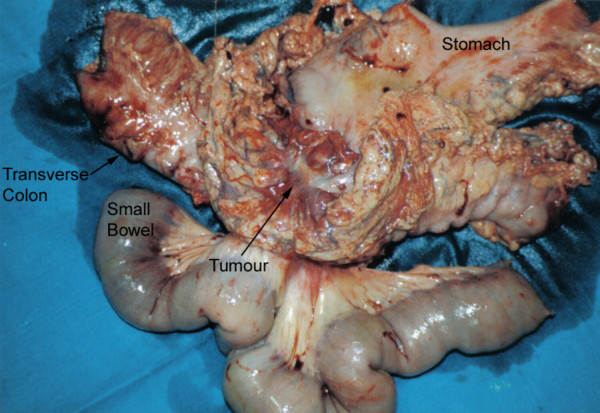
Macroscopic en-bloc surgical specimen showing fistula between stomach and transverse colon (arrowed).

In view of her symptoms, an exploratory laparotomy was undertaken. At surgery, a large mobile tumour of the distal transverse colon was identified; this was adherent to the greater curvature of the stomach, the mesentery and to several loops of jejunum. A radical *en-bloc *resection was performed involving a subtotal gastrectomy, transverse colectomy and small bowel resection (Figure 2). The patient made an uneventful recovery from surgery. Histology revealed a poorly differentiated mucinous adenocarcinoma of colon without lymphatic involvement (Dukes' Stage B): this was adherent to and had penetrated the stomach wall. She received adjuvant 5-fluorouracil (420 mg/m^2^) and folinic acid (20 mg/m^2^) chemotherapy every four weeks for the following six months.

She has been followed-up with two-yearly colonoscopy and five-yearly upper gastrointestinal endoscopy. She remains well with no signs of either local or distant recurrence more than ten years after initial diagnosis.

## Discussion

Advanced neoplasms of the stomach and transverse colon are the commonest causes of a gastrocolic fistula: adenocarcinoma of the transverse colon is commoner in the Western World [1,3,4], whereas adenocarcinoma of the stomach is a more frequent cause in Japan [5]. Gastrocolic fistula has also been reported with other tumour types such as gastric lymphoma [7], carcinoid tumours of the colon [8] and rarely, metastatic tumours [9] and infiltrating tumours of the pancreas, duodenum and biliary tract [3]. With advances in medical treatment, gastrocolic fistula secondary to peptic ulcer disease is now less common [6]. A variety of other causes of gastrocolic fistula have been reported: these include syphilis, tuberculosis, abdominal trauma, Crohn's disease, Cytomegalovirus gastric infection in AIDS patients and percutaneous endoscopic gastrostomy (PEG) tubes [10-13].

The fistulous connection in a gastrocolic fistula usually arises between the greater curvature of the stomach and the distal half of the transverse colon because of their close anatomical proximity separated only by the gastrocolic omentum [13]. Two theories have been advanced for the development of a fistula [1,3,4]: the tumour may invade directly across the gastrocolic omentum from the orginating organ; alternatively, a tumour ulcer may provoke a surrounding inflammatory peritoneal reaction leading to the adherence and fistulation between the two organs. Cases of malignant gastrocolic fistula have usually been characterised by the presence of large infiltrative tumours with a surrounding inflammatory reaction, as seen in our patient; lymph node involvement is unusual [13].

Our patient presented with the characteristic triad of symptoms associated with a gastrocolic fistula [5,14]: diarrhoea, weight loss and faeculent vomiting. Other symptoms include: abdominal pain, fatigue, faeculent eructations and nutritional deficiencies. The gastrocolic fistula was identified in our patient by means of an upper gastrointestinal contrast series. Because the flow in the fistula is predominantly from transverse colon to stomach [15], several authors have suggested that barium enema is the more sensitive investigation in detecting and delineating such a fistula, although the detection rate may be lower in neoplastic cases [2,16-18]. Computerised tomography may also be useful in both delineating the fistula and identifying the underlying aetiology [5,19]. Endoscopy is an excellent tool for visualising the fistulous opening (especially in the stomach) and also allows preoperative histological confirmation [20,21].

Although two stage approaches have been advocated historically for malignant gastrocolic fistula, in order to first correct nutritional deficiencies [22], most authors now prefer radical en-bloc resections [14]. Despite such approaches, most patients have a poor prognosis and no patient has survived for more than nine years after resection [5]. This case report describes the longest disease free survival of a patient with a malignant gastrocolic fistula. To the authors' knowledge, she is also the youngest patient to be reported. It is worth noting that colorectal cancer in patients aged less than 35 years is normally associated with a poorer prognosis compared with older age groups [23-25]. This is related to the biological characteristics of such tumours with a higher proportion of mucinous poorly differentiated tumours. As a result, younger patients present with more advanced disease. Such patients require early diagnosis and a radical approach to treatment.

## Conclusions

Gastrocolic fistula is an uncommon presentation of adenocarcinoma of the transverse colon. Radical en-bloc surgery with adjuvant chemotherapy may occasionally produce long-term survival.

## Competing interests

The author(s) declare that they have no competing interests.

## Authors' contributions

**MJF **collated the information, searched literature and wrote the manuscript.

**JKD **assisted in literature search and writing of the manuscript.

**KM **was responsible for long-term follow up of the patient and assisted in literature search.

**MCP **managed the patient, helped in preparing the manuscript and edited the final version.

All authors have read and approved the final version of the manuscript.
